# Autocharging Techniques for Implantable Medical Applications

**DOI:** 10.1155/2021/6074657

**Published:** 2021-10-19

**Authors:** Hamza Abu Owida, Jamal I. Al-Nabulsi, Nidal M. Turab, Feras Alnaimat, Hana Rababah, Murad Y. Shakour

**Affiliations:** ^1^Medical Engineering Department, Faculty of Engineering, Al-Ahliyya Amman University, Amman 19328, Jordan; ^2^Department of Networks and Information Security, Faculty of Information Technology, Al-Ahliyya Amman University, Amman 19328, Jordan; ^3^Electrical Engineering Department, Faculty of Engineering, Al-Ahliyya Amman University, Amman 19328, Jordan

## Abstract

Implantable devices have successfully proven their reliability and efficiency in the medical field due to their immense support in a variety of aspects concerning the monitoring of patients and treatment in many ways. Moreover, they assist the medical field in disease diagnosis and prevention. However, the devices' power sources rely on batteries, and with this reliance, comes certain complications. For example, their depletion may lead to surgical interference or leakage into the human body. Implicit studies have found ways to reduce the battery size or in some cases to eliminate its use entirely; these studies suggest the use of biocompatible harvesters that can support the device consumption by generating power. Harvesting mechanisms can be executed using a variety of biocompatible materials, namely, piezoelectric and triboelectric nanogenerators, biofuel cells, and environmental sources. As with all methods for implementing biocompatible harvesters, some of them are low in terms of power consumption and some are dependent on the device and the place of implantation. In this review, we discuss the application of harvesters into implantable devices and evaluate the different materials and methods and examine how new and improved circuits will help in assisting the generators to sustain the function of medical devices.

## 1. Introduction

As technology progresses, especially in the biomedical domain, implantable devices are becoming more sophisticated in their efficiency and physical dimensions. Currently, biomedical implantable devices play an important role in a variety of medical aspects such as diagnosis and treatment. These devices can monitor various diseases using efficient circuit designs and biocompatible materials in collaboration with high resolution biosensors [1–4].

The biomedical implantable devices referred to are subcutaneous devices, namely, defibrillators, pacemakers, drug pumps, cochlear implants, and stimulators. For further illustration of the impact of implantable devices, we consider patients who suffer from an irregular heartbeat (arrhythmia) and have to be monitored and analyzed regularly. With the assistance of a pacemaker, patients suffering from arrhythmia can live a normal life. Additionally, nonimplantable devices that are readily available on the market, such as watches and rings that assist in the monitoring aspect, can provide great assistance with the mentioned biomedical devices [5–7].

Although implantable medical devices can be very reliable and generally suffer only minor complications, they have a major drawback when it comes to powering them. Implantable medical devices are mainly electronic circuits that rely completely on batteries to sustain their function. However, batteries eventually run out of power and, in this case, require surgical intervention in order to replace them. Furthermore, the aim of nearly all implantable devices is to reduce the space they take and not be constantly felt by the patient. With current technology, this aim is plausible, but made difficult due to the batteries' limited power storage capacity; there is, therefore, a desperate need to find ways to harvest power for the batteries from the human body or its internal surroundings [1–3, 7].

A typical implantable medical device does not require a large amount of power to function. The amount of power needed depends on the device's function and voltage specifications, which are usually as little as 2-3 volts [5]. The two most important aspects to consider are the power consumption and generation: the goal is to minimize the power consumption within the device circuit components and to realize a way to increase device efficiency in the power generation, thereby increasing the lifespan of the device. However, it is essential to understand the power needed by a certain device in order to ensure the most suitable adjustments [1, 5].

## 2. Piezoelectric-Based Energy Harvesting

Mechanical energy to electrical energy conversion is the core concept of lead zirconate titanate (PZT) energy harvesting. If an exterior load is applied to piezoelectric material, it will cause the piezoelectric capacity to be assigned to a polar surface's dipole charges [8].

The PZT conversion efficiency is determined by the characteristics of the PZT substance. The first *in vivo* implanted PZT as energy harvester was developed in 1980 [5]. So far, PZT and polyvinyl fluoride (PVDF) are among the most popular piezoelectric materials [5, 9, 10].

**Figure 1:**
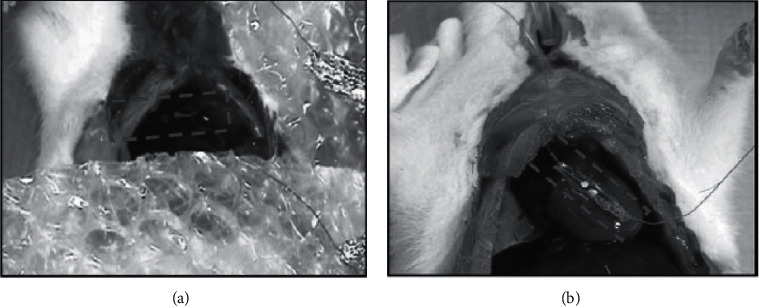
Breath and heartbeat energy harvesting of a live rat using a PZT. A PZT attached to the live rat's chest (a) and its heart (b), which drives the PZT to intermittently twist and harvest an AC power yield.

Wang and Song implemented ZnO-nanowired piezoelectric nanogenerator to harvest small amounts of vibrational energy. The performance of the ZnO nanowire PENG energy harvester was evaluated as being around 10 pW/*μ*m^2^ (in terms of power density). This model has the ability to transform vibrational energy into electricity, which could be used to drive nanodevices [11]. Li et al. demonstrated an implanted PENG due to a sole ZnO nanowire. This system was connected to a living rat's diaphragm and heart to extract energy from breathing and heartbeat, and the typical harvest voltage was less than 50 mV [12]. Dagdeviren et al. introduced a template of PENG with a flake structure as a PZT set (Figure 1) to extract energy from living organs, such as the heart and lungs, in an animal model, with their energy harvester estimated at around 0.18 *μ*W/cm^−2^ [13].

Cheng et al. employed an ultrathin membrane of PVDF piezoelectric to wrap around the aortic valve of a pig, extracting a maximum yield power of 40 nW *in vitro* through transforming the aorta's expansion and retraction mechanism [14]. Hwang et al. designed a PZT energy harvester based on crystalline ultrathin PZT film, directly stimulating a rat's heart through two metal wires. During one operating cycle, the system produced 2.7 *μ*J of peak energy, which is greater than the threshold energy needed to cause the action potential of a heart contraction, approximately 1 *μ*J [15]. In 2017, Jeong et al. developed a PZT energy harvester based on a thin lead-free film, which was sutured on a pig's heart and driven by the pig's heartbeat, capable of producing 5 V [16]. Han's team proposed a three-dimensional PZT micromodel as a thin film of PVDF, which was capable of extracting kinetic energy from the mouse's hind leg and producing an output voltage of around 0.79 V [17]. In 2020, Xu et al. developed a tubular microchip composite that fits on the pacemaker electrode and extracts energy from the dynamic motion of a heartbeat with a measured average output peak voltage of 3.22 V [18].

## 3. Triboelectric Nanogenerator-Based Energy Harvesting

**Figure 2:**
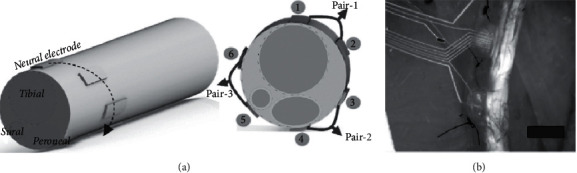
(a) Diagram of location and structure of a sling interface embedded on a sciatic nerve: pair 1 (active electrodes 1 and 2), pair 2 (active electrodes 3 and 4), and pair 3 (active electrodes 5 and 6). (b) Live image of the inserted sling interface on the sciatic nerve; the scale bar is 2 mm.

The triboelectric nanogenerator (TENG) is based on the triboelectrification and electrostatic induction coupling properties. The surfaces of the two friction layers are charged with triboelectric effects as they come into contact due to the triboelectrification influence [1, 5, 19]. Zheng et al. developed a TENG energy harvester based on polydimethylsiloxane (PDMS) film that was inserted beneath the rat's left chest skin. Then, the vibrational breathing energy was explicitly converted to electricity with an estimated power density of 8.44 mW/m^2^ and could be used to drive a pacemaker model [20]. In 2018, Jiang et al. implanted a bioabsorbable TENG in a rat's dorsal hypodermic area, and the evaluated power density reached 21.6 mW/m^2^ [21]. In 2018, Li et al. developed a set of bioresorbable TENGs supplemented with gold nanorods that were inserted in the subcutaneous regions on rats' backs. Subsequently, it has been determined that TENG biological decay *in vivo* can indeed be photothermally modified to the photocatalytic activity of the gold nanorods. The maximum output voltage *in vivo* harvest electrical power was 2 V. Furthermore, an *in vivo* output voltage was applied to fibroblast cells, resulting in a major acceleration of wound healing, and it was very effective to the recovery process [22]. Lee et al. in 2018 used a water/air integrated TENG for peripheral nerve stimulation to modulate the rat's leg muscles (Figure 2) and achieve a peak power value of 2.93 W [23].

Yao et al. developed a completely inserted TENG for vagus nerve stimulation in rats that was applied to the rat's stomach's surface and produced an electric pulse during stomach activity with a maximum output power up to 40 *μ*W [24]. Wang et al. designed a layered diode amplified TENG powered by hand beating *in vitro* for stimulation of a rat's leg muscle with a peak power of 500 *μ*W [25]. In 2020, Wu and coresearchers developed a skin-like liquid solitary electrode based on TENG that is sustainable and flexible, performing various body movements such as folding, spinning, crumpling, and stretching, with a maximum peak power density of 4.61 W/m^2^ [26].

## 4. Biochemical Energy Harvesting

**Figure 3:**
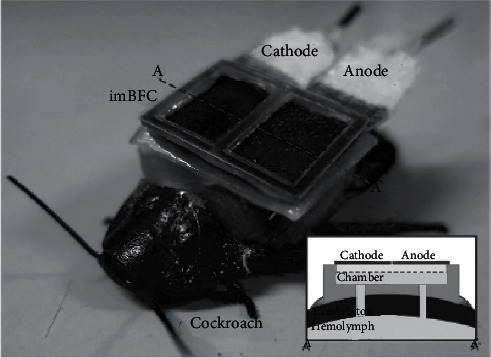
Photograph of a cockroach wearing implanted biofuel cells.

The technique of biochemical renewable energy involves *in vivo* research to generate power and was first introduced in 1974 for medical implanted devices approaches [1, 5]. Biofuel cells generate electricity using biocatalysts. There are infinite amounts of biofuels in living organisms that further produce microwatts of power, which cells are using in biochemical reactions to transform biochemical energy into electric energy [27]. Zhao et al. in 2009 compared mesoporous carbons and carbon nanotube biofuel cells in the electric output efficiency, which produced a maximum power density of 38.7 *μ*W/cm^2^ and 2.1 *μ*W/cm^2^, respectively [28]. Miyake et al. in 2011 proposed a miniature assembly with a bioanode needle for accessing biofuels in a rabbit ear's artery, yielding a maximum power of 26.5 mW [29]. Rasmussen et al. harvested energy from a disaccharide trehalose biofuel cell implanted in *Blaberus discoidalis* via cuts inside its abdomen, and the generated output power density was 55 *μ*W/cm^2^ [30]. Shoji et al. in 2016 examined enzymatic biofuel cells with cockroaches (Figure 3), which are capable of delivering a 333 *μ*W yielded power and powering both of a light-emitting diode and a wireless temperature sensor [31].

## 5. Environmental Energy Harvesting

Bioenvironmental energy harvesting is a method of driving implantable medical devices by utilizing a variety of low-grade power sources, such as ultrasonic transducers, electromagnetic generators, and photovoltaic panels [1, 32]. Various studies have been carried out using ultrasonic transducers as energy harvesters to power implantable medical devices. Kawanabe et al. embedded a piezoelectric oscillator beneath a goat's skin to receive vibrational energy created by an ultrasonic transducer placed exterior to the goat's skin; the measured transmission output power was 2.1 W from the presented model [33]. Tower *et al.* introduced a system that could be used for biological evaluation and monitoring. The energy of a surface-applied ultrasound beam was converted to a high-frequency current by this device [34]. In 2019, Alam et al. assembled a PENG using an implantable piezoelectric with BaTiO_3_ to stimulate a rat's paretic muscles actively with endogenous ultrasonic acoustic energy, and the stimulated maximum power was 5.95 mW [35]. Kim et al. proposed an ultrasonically implantable model microlight for cancer therapy based on piezoelectric materials to turn on a surface-mount light-emitting diode with a range of power density of 0.048–6.5 mW/cm^2^ [36]. The human body produces an abundance of kinetic renewable resources during body movement; in this model, the mechanical transmission mechanism will transform body movement into rotating activity, which is then used to wind a spring to generate mechanical energy. When a certain amount of mechanical energy is achieved, the spring allows the electromagnetic generator to generate electrical pulses [1, 32].

Goto et al. designed an automated quartz watch to transform heartbeat power into electricity, which was applied to a dog's right ventricular wall. The 80 mJ transmit power was deposited in a capacitor and used to power a pulse generator circuit that paced the dog's heart for 30 minutes [37]. Zurbuchen et al. designed an automated wristwatch that transformed mechanical heartbeat energy to electrical energy and was grafted into a sheep's heart. The proposed model could produce 16.7 *μ*W of power [38]. Automatic wristwatches were designed by Zurbuchen's team to be a set of power sources implanted on the hearts of living pigs, and the average power yield was 50 *μ*W [39]. In 2019, Haeberlin et al. designed an electromagnetic generator with biocompatible cardiac turbines to extract energy from blood circulation that could be then inserted into the pig's heart via catheter; in addition, light energy harvesting has been used to operate implantable devices [40].

Photovoltaic cells are implanted to transform sunlight energy to electrical energy. Additionally, semiconductors are widely used to produce voltages and current as a function of optical emission by the photovoltaic effect [5]. Laube et al. inserted a photovoltaic cell set and an LED to a rabbit's eye creating a biopsied energy harvesting device powered by light with a 1.5 mW power yield [41].

**Figure 4:**
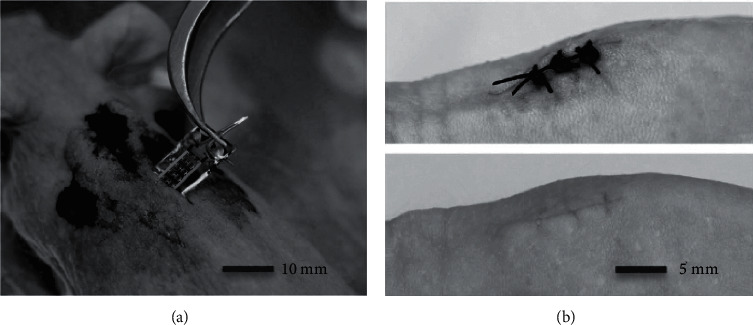
Clinical processes and electrical interpretation of the subdermal photovoltaic devices in vivo. (a) Live image through surgical process to insert the embedded photovoltaic device beneath the mouse's back skin. (b) Optical images of the clinical wound after sewing (upper) and after healing (lower).

Haeberlin et al. embedded a photovoltaic cell in a pig's transcutaneous site to harvest optical energy and operate a peacemaker constantly, at a rate up to 125 bpm for 2 weeks in darkness [42]. Song et al. documented solar cell arrays in a hairless mouse's transcutaneous site (Figure 4) and harvested electrical power up to 647 *μ*W [43]. In 2018, Wu et al. developed an implanted chip comprising photovoltaic panels as a 256-pixel artificial subretina that was mounted in the subsequent pole of a pig's eyeball with maximum output converted current up to 16.7 *μ*A [44].

## 6. Conclusions

Although implantable devices have dramatically increased in their efficiency as a result of their batteries having more storage capabilities and the circuits within the average implantable device consuming less power, there is still a great challenge in finding ways to harvest power back to their battery packs. Patients who use implantable devices still suffer from the fact that the batteries have to be changed, requiring potentially costly surgical interventions. To reduce the need for surgery, the harvesting technique regarding the implantable device is a potentially fruitful approach, whether to eliminate the use of batteries entirely or—in the case of recharging them—to delay the need for surgical intervention. Several harvesting methods have been presented in this review; they were nominated due to their sensible application with regard to their power output, advantages, and disadvantages with the illustration of their testing on animals and humans.

A major challenge with implantable devices is to reduce the feeling of their existence within the bearer; therefore, it is preferable that the devices are smaller and more efficient. Since batteries cause implantable devices to become bulkier, it is recommended that they become smaller or eliminated. Moreover, implantable devices do not require large amounts of energy (usually 2-3 volts) to function, and looking for methods of harvesting power may be more plausible than having a battery with toxic materials that may leak into the body of the patient.

Although the mentioned techniques are promising when it comes to the harvesting of power, some are more preferable than others and are more likely to be applicable regarding safety and durability in the real world. For instance, techniques using piezoelectric harvesters cannot be applied in the majority of the implantable devices since they generate electricity by applying force or pressure and they have to be inserted next to kinetic living organs such as the lungs and heart. Even though they are very safe and biocompatible, their electricity harvesting is not up to par regarding their size and space needed. Similarly, biofuel cells have many of the same features and produce the same amount of power while taking up less space in the body. Furthermore, many of these techniques were studied *in vivo* for less than one year, with additional research being required to extend to long-term *in vivo* performance. A key challenge is determining how to maintain stable system performance and ensure excellent biocompatibility during long-term *in vivo* implantation under complex conditions. Another downside of the mentioned techniques is the need to transfer harvested energy from the harvester to the biomedical sensor without laying exterior wires or conductors for the sake of the patients' safety and comfort. This requirement is not yet investigated or studied thoroughly.

The extant energy harvesting techniques for implantable devices are promising. However, due to the presence of certain drawbacks and disadvantages that might delay their application and installment in implantable devices commercially, it is crucial to understand every method thoroughly. By comparing multiple techniques, the advantages may be merged together, and downsides in one technique can be compensated for using the advantages of another one. Furthermore, enhancements could be made to the components leading to less energy consumption, with existing harvesting techniques becoming sufficient enough for future applications and perhaps allowing the elimination of batteries altogether.

Nevertheless, given that all energy harvesters have drawbacks, we suggest a hybrid harvester to address these issues. Hybrid energy harvesters are capable of scavenging power from different sources and thus offset any restrictions due to the lack of one energy source. If one or more energy sources are accessible, a hybrid device is guaranteed to receive energy. A further incorporation of advanced engineering principles with existing power harvesters offers guidance for future studies. The combination of different energy harvesters and advanced nanotechnology can lead to solutions to long-term obstacles and can open the way for the development of new technologies for disease detection, treatment, and prevention.  Table 1 summarizes the highest power outputs obtained in the discussed studies.

A final observation from Table 1 arises when comparing the harvested energy from the studied technologies. The biochemical energy harvester produces maximum power and power density, while environmental energy harvesters produce maximum current and energy. Finally, piezoelectric-based energy harvesters produce maximum voltage.

## Figures and Tables

**Table 1:** Highest power outputs given in reviewed works.

Energy harvesting technology	Power (W), power density (W/m^2^), voltage (V)	Reference
Piezoelectric-based energy harvesting	0.18 *μ*W/cm^2^	[13]
	0.1 mJ	[15]
	5 V	[16]
Triboelectric nanogenerator-based energy harvesting	2 V	[22]
	2.93 W	[23]
	416 *μ*W/cm^2^	[26]
Biochemical energy harvesting	1 mW	[29]
	402 mW/m^2^	[30]
Environmental energy harvesting	5.95 mW	[35]
	80 mJ	[37]
	16.7 *μ*A	[44]

## References

[1] Jiang D., Shi B., Ouyang H., Fan Y., Wang Z. L., Li Z. (2020). Emerging implantable energy harvesters and self-powered implantable medical electronics. *ACS Nano*.

[2] Gibney E. (2015). The inside story on wearable electronics. *Nature News*.

[3] Hinchet R., Kim S. W. (2015). Wearable and implantable mechanical energy harvesters for self-powered biomedical systems. *ACS Nano*.

[4] Dagdeviren C., Li Z., Wang Z. L. (2017). Energy harvesting from the animal/human body for self-powered electronics. *Annual Review of Biomedical Engineering*.

[5] Zhao J., Ghannam R., Htet K. O. (2020). Self‐Powered implantable medical devices: photovoltaic energy harvesting review. *Advanced Healthcare Materials*.

[6] Das R., Moradi F., Heidari H. (2020). Biointegrated and wirelessly powered implantable brain devices: a review. *IEEE Transactions on Biomedical Circuits and Systems*.

[7] Shi B., Li Z., Fan Y. (2018). Implantable energy‐harvesting devices. *Advanced Materials*.

[8] Lim Y. Y. Piezoelectric based energy harvesting from human motions using double pendulum system.

[9] Gupta S., Fatma B., Bhunia R., Gupta R. K., Garg A. (2021). *Biomechanical Energy Harvesting with Piezoelectric Materials*.

[10] Wan C., Bowen C. R. (2017). Multiscale-structuring of polyvinylidene fluoride for energy harvesting: the impact of molecular-, micro-and macro-structure. *Journal of Materials Chemistry*.

[11] Wang Z. L., Song J. (2006). Piezoelectric nanogenerators based on zinc oxide nanowire arrays. *Science*.

[12] Li Z., Zhu G., Yang R., Wang A. C., Wang Z. L. (2010). Muscle‐driven in vivo nanogenerator. *Advanced Materials*.

[13] Dagdeviren C., Yang B. D., Su Y. (2014). Conformal piezoelectric energy harvesting and storage from motions of the heart, lung, and diaphragm. *Proceedings of the National Academy of Sciences*.

[14] Cheng X., Xue X., Ma Y. (2016). Implantable and self-powered blood pressure monitoring based on a piezoelectric thinfilm: simulated, in vitro and in vivo studies. *Nanomaterials and Energy*.

[15] Hwang G. T., Park H., Lee J. H. (2014). Self‐powered cardiac pacemaker enabled by flexible single crystalline PMN‐PT piezoelectric energy harvester. *Advanced Materials*.

[16] Jeong C. K., Han J. H., Palneedi H. (2017). Comprehensive biocompatibility of nontoxic and high-output flexible energy harvester using lead-free piezoceramic thin film. *APL Materials*.

[17] Han M., Wang H., Yang Y. (2019). Three-dimensional piezoelectric polymer microsystems for vibrational energy harvesting, robotic interfaces and biomedical implants. *Nature Electronics*.

[18] Xu Z., Jin C., Cabe A. (2020). Flexible energy harvester on a pacemaker lead using multibeam piezoelectric composite thin films. *ACS Applied Materials & Interfaces*.

[19] Wang L., Liu W., Yan Z., Wang F., Wang X. (2021). Stretchable and shape‐adaptable triboelectric nanogenerator based on biocompatible liquid electrolyte for biomechanical energy harvesting and wearable human–machine interaction. *Advanced Functional Materials*.

[20] Zheng Q., Shi B., Fan F. (2014). In vivo powering of pacemaker by breathing‐driven implanted triboelectric nanogenerator. *Advanced Materials*.

[21] Jiang W., Li H., Liu Z. (2018). Fully bioabsorbable natural‐materials‐based triboelectric nanogenerators. *Advanced Materials*.

[22] Li Z., Feng H., Zheng Q. (2018). Photothermally tunable biodegradation of implantable triboelectric nanogenerators for tissue repairing. *Nanomaterials and Energy*.

[23] Lee S., Wang H., Wang J. (2018). Battery-free neuromodulator for peripheral nerve direct stimulation. *Nanomaterials and Energy*.

[24] Yao G., Kang L., Li J. (2018). Effective weight control via an implanted self-powered vagus nerve stimulation device. *Nature Communications*.

[25] Wang H., Wang J., He T., Li Z., Lee C. (2019). Direct muscle stimulation using diode-amplified triboelectric nanogenerators (TENGs). *Nano Energy*.

[26] Wu Y., Luo Y., Qu J., Daoud W. A., Qi T. (2020). Sustainable and shape-adaptable liquid single-electrode triboelectric nanogenerator for biomechanical energy harvesting. *Nano Energy*.

[27] Xu C., Song Y., Han M., Zhang H. (2021). Portable and wearable self-powered systems based on emerging energy harvesting technology. *Microsystems & Nanoengineering*.

[28] Zhou M., Zhai Y., Dong S. (2009). Electrochemical sensing and biosensing platform based on chemically reduced graphene oxide. *Analytical Chemistry*.

[29] Miyake T., Haneda K., Nagai N. (2011). Enzymatic biofuel cells designed for direct power generation from biofluids in living organisms. *Energy & Environmental Science*.

[30] Rasmussen M., Ritzmann R. E., Lee I., Pollack A. J., Scherson D. (2012). An implantable biofuel cell for a live insect. *Journal of the American Chemical Society*.

[31] Shoji K., Akiyama Y., Suzuki M., Nakamura N., Ohno H., Morishima K. (2016). Biofuel cell backpacked insect and its application to wireless sensing. *Biosensors and Bioelectronics*.

[32] Hannan M. A., Mutashar S., Samad S. A., Hussain A. (2014). Energy harvesting for the implantable biomedical devices: issues and challenges. *Biomedical Engineering Online*.

[33] Kawanabe H., Katane T., Saotome H., Saito O., Kobayashi K. (2001). Power and information transmission to implanted medical device using ultrasonic. *Japanese Journal of Applied Physics*.

[34] Towe B. C., Larson P. J., Gulick D. W. Wireless ultrasound-powered biotelemetry for implants.

[35] Alam M., Li S., Ahmed R. U. (2019). Development of a battery-free ultrasonically powered functional electrical stimulator for movement restoration after paralyzing spinal cord injury. *Journal of Neuroengineering and Rehabilitation*.

[36] Kim A., Zhou J., Samaddar S. (2019). An implantable ultrasonically-powered micro-light-source (*μ*Light) for photodynamic therapy. *Scientific Reports*.

[37] Goto K., Nakagawa T., Nakamura O., Kawata S. (2001). An implantable power supply with an optically rechargeable lithium battery. *IEEE Transactions on Biomedical Engineering*.

[38] Zurbuchen A., Pfenniger A., Stahel A. (2013). Energy harvesting from the beating heart by a mass imbalance oscillation generator. *Annals of Biomedical Engineering*.

[39] Zurbuchen A., Haeberlin A., Pfenniger A. (2016). Towards batteryless cardiac implantable electronic devices—The swiss way. *IEEE Transactions on Biomedical Circuits and Systems*.

[40] Haeberlin A., Rösch Y., Tholl M. V. (2019). Intracardiac turbines suitable for catheter-based implantation—an approach to power battery and leadless cardiac pacemakers?. *IEEE Transactions on Biomedical Engineering*.

[41] Laube T., Brockmann C., Buß R. (2004). Optical energy transfer for intraocular microsystems studied in rabbits. *Graefe’s Archive for Clinical and Experimental Ophthalmology*.

[42] Haeberlin A., Zurbuchen A., Walpen S. (2015). The first batteryless, solar-powered cardiac pacemaker. *Heart Rhythm*.

[43] Song K., Han J. H., Lim T. (2016). Subdermal flexible solar cell arrays for powering medical electronic implants. *Advanced Healthcare Materials*.

[44] Wu C.-Y., Kuo P.-H., Lin P.-K. (2018). A CMOS 256-pixel photovoltaics-powered implantable chip with active pixel sensors and iridium-oxide electrodes for subretinal prostheses. *Sensors and Materials*.

